# Assessing the intake of obesity-related foods and beverages in young children: comparison of a simple population survey with 24 hr-recall

**DOI:** 10.1186/1479-5868-6-71

**Published:** 2009-10-26

**Authors:** Cheryl-Ann Bennett, Andrea M de Silva-Sanigorski, Melanie Nichols, Andrew C Bell, Boyd A Swinburn

**Affiliations:** 1 WHO Collaborating Centre for Obesity Prevention, Deakin University, Geelong 3217, Australia; 2 University of Newcastle, Callaghan, NSW 2308, Australia

## Abstract

**Background:**

With an increasing focus on obesity prevention there is a need for simple, valid tools to assess dietary indicators that may be the targets of intervention programs. The objective of this study was to determine the relative validity of previous day dietary intake using a newly developed parent-proxy questionnaire (EPAQ) for two to five year old children.

**Methods:**

A convenience sample of participants (n = 90) recruited through preschools and the community in Geelong, Australia provided dietary data for their child via EPAQ and interviewer-administered 24-hour dietary recall (24 hr-recall). Comparison of mean food and beverage group servings between the EPAQ and 24 hr-recall was conducted and Spearman rank correlations were computed to examine the association between the two methods.

**Results:**

Mean servings of food/beverage groups were comparable between methods for all groups except water, and significant correlations were found between the servings of food and beverages using the EPAQ and 24-hr recall methods (ranging from 0.57 to 0.88).

**Conclusion:**

The EPAQ is a simple and useful population-level tool for estimating the intake of obesity-related foods and beverages in children aged two to five years. When compared with 24-hour recall data, the EPAQ produced an acceptable level of relative validity and this short survey has application for population monitoring and the evaluation of population-based obesity prevention interventions for young children.

## Background

The increasing prevalence of obesity in children has been partially attributed to the over-consumption of energy-dense, nutrient poor foods and sugar-sweetened beverages [1-6]. Fast food, packaged snacks, sweet drinks, and biscuits (cookies) have also been shown to contribute significant amounts of energy to children's diets in Australia [7,8], as in other Western countries.

In general, dietary assessment tools for children assess the whole diet and are focused on individual nutritional adequacy or excess. There is, however, a demand for validated tools that specifically assess the intake of energy dense foods and sugar sweetened beverages which are easily and cheaply administered to large groups for population monitoring and the evaluation of obesity-related health promotion programs. Such tools are particularly lacking for use in populations of young children [9,10] which requires proxy reporting from parents, guardians or caregivers [11], a method which, despite having some limitations [12], has been shown to be valid [11,13-17].

The advantages of using short food frequency questionnaires include reduced costs and participant burden, reduced data handling and processing, and the ability to collect cross-sectional data more frequently and/or from a larger sample of participants for population monitoring purposes [18-21]. The purpose of this study was to conduct a relative validity analysis of dietary questions within a short Eating and Physical Activity Questionnaire (EPAQ) compared with a 24-hour dietary recall (24 hr-recall), with the aim of assessing the ability of the questionnaire to determine consumption of obesity-related foods and beverages by two to five year old Australian children.

## Methods

Participants were volunteers recruited through preschools (four-year-old kindergarten), word of mouth and study flyers throughout the City of Greater Geelong, Australia. Participants recruited through preschools were interviewed on-site and all others attended Deakin University for the interview. All participants were parents of children aged two to five years and all provided informed consent to record the session. The study was approved by the Deakin University Human Research Ethics Committee (EC 21-2006).

### Procedure

The parent or guardian was asked to complete the EPAQ followed by a 'triple pass' 24 hr-recall interview. The triple pass method is designed to maximise the ability of respondents to recall what was eaten and drunk and has been shown to aid in the recollection of dietary intake through time and place associations [22,23]. The 24 hr-recall included all meals, snacks and beverages consumed and consisted of three sections: 1) a quick list of foods and beverages consumed; 2) details of type, amount, cooking method and time of consumption; and 3) recall-review allowing participants to report items that may have previously been forgotten, state whether intake was typical and report usage of dietary supplements.

Interviews were conducted by trained researchers and final year dietetic students (in pairs) and the order of administration of the two recall methods was deliberately not randomized to ensure that parents providing responses to the EPAQ would not be sensitized to recall their child's diet by having previously completed a detailed 24 hr-recall. In general, the questionnaire and interview took a total of approximately 30 minutes to complete. Interviews captured both weekdays and weekend days.

### Eating and Physical Activity Questionnaire (EPAQ)

The questionnaire contains items about demographic characteristics, activity levels and dietary information and took five to ten minutes to complete. An initial draft of the EPAQ was piloted before use in the community with a convenience sample of parents (n = 15) and Maternal and Child Health Nurses (n = 4). Small adjustments were made to wording and layout of the survey in response to discussion and feedback about the survey, in order to improve clarity and appropriateness of questions. This paper focuses only on the intakes of the ten food and beverage categories (fruit juice, sugar-sweetened drinks [including carbonated soft drinks and cordial, a sugar syrup that provides approximately 160 kJ per 100 ml when diluted with water according to directions], water, plain milk, flavoured milk, vegetables, packaged snacks, fruit, chocolate and confectionary, and cake and sweet biscuits). Dietary questions focused on gathering food and beverage intake data from 'yesterday', the day prior to the interview.

Parents were asked to complete the EPAQ independently, but with with the aid of a 'Food Servings Guide' which provided pictures to assist in the estimation of serving sizes of foods and drinks included in the EPAQ (survey and guide available at: ). The dietary questions required categorical responses and focused on 'key foods' with positive or negative associations with body weight [7,24-26]. The response categories varied and the options for beverages ranged from zero, 1/2 a serving then in whole numbers of servings up to five or more. All beverages had a serving size of 125 ml (1/2 cup), while the servings for the food categories varied but were clearly stated and pictured in the Food Servings Guide. All food serving sizes were based on the recommended servings in the Australian Guide to Healthy Eating (AGHE) [27].

### 24-hour dietary recall

Parents were asked to describe their child's routine during the previous day followed by a 'triple pass' 24 hr-recall of all foods and beverages their child consumed. This procedure was based on that used by the Australian Bureau of Statistics in the 1995 National Nutrition Survey, and is designed to maximize the ability of respondents to recall what was consumed. It was also more recently used in the 2007 National Children's Nutrition and Physical Activity Survey [22]. Household measures (measuring cups, plates, bowls and glasses) were used to help parents to estimate food portion sizes.

### Data Handling

Data was double entered into EpiData (2006, EpiData Association, Denmark) and transferred to Stata SE v10 (StataCorp, Texas USA) for data management, cleaning and analysis. No extreme or implausible data were identified and all cases were included in subsequent analysis.

#### Transposition of Foods from Dietary Recall

Food and beverage intakes from the 24 hr-recall were coded according to the categories and serving sizes on the questionnaire for ease of comparison. For example a 250 ml glass of juice = two servings, one apple = one serving of fruit, and 1/2 cup vegetables = one serving. This data were also entered into FoodWorks Professional (2005, Xyris Software, Queensland, Australia) to obtain energy content of the food and beverage categories for calculation of energy per serving and energy density. The volumes of food and beverages, in grams or millilitres, from the 24 hr-recall were converted into servings using reference serving sizes from the AGHE [27], except for beverage serving sizes which were 125 ml per serving. Food category serving sizes were: fruit = 150 g, vegetables = 75 g, chocolate and confectionary = 25 g, cake and sweet biscuits = 40 g and packaged snacks = 25 g.

Socio-demographic information was obtained from the questionnaire and socioeconomic status (SES) was classified using quartiles of the Socio-Economic Index for Areas (SEIFA), index of relative socio-economic advantage-disadvantage based on the postcode of the participant's home. This area-level indicator was derived from the 2001 census and assigns an index to postal areas based on socioeconomic variables such as education, occupation, family structure and ethnicity [28].

### Statistical Analysis

All food and beverage groups were non-normally distributed and the Kruskal-Wallis non-parametric test of differences in means was used to test for significant differences between the two methods. Spearman correlations were used to estimate the degree of association between methods. Statistical analysis was conducted using Stata SE v10 and in all cases p < 0.05 was considered statistically significant.

## Results

Demographic characteristics for the 90 children were as follows; the mean age was 3.98 ± 0.98 years, 56% (n = 50) were female, and on the day of interest ('yesterday') 55 children attended some form of care outside the home. A high percentage of the children's parents were university educated (41% of mothers and 38% of fathers), 63% of families were in the highest two quartiles of SES, and 87% were two parent households. The majority of respondents (95%) were the child's mother.

Consumption of food and beverages for all 10 categories according to both data collection methods are shown in Table 1. Servings were comparable between the two methods for all food categories and all beverage groups other than water, which was significantly lower using the EPAQ. There was no difference in the proportion of children reported to have no (zero) intake of a particular food/beverage group between the two methods. The servings for all food and drink categories were significantly correlated between the two methods (Spearman correlations ranged from 0.57 to 0.88, p < 0.001).

**Table 1:** Comparison of servings of food and beverage categories by the Eating and Physical Activity Questionnaire (EPAQ) and 24 hr-recall methods (n = 90)

	**EPAQ**		**24 hr-recall**			
**Food/Beverage groups**	**Consumption %**	**Servings Mean (95% CI)**	**Consumption %**	**Servings Mean (95% CI)**	**Significance (p-value)**^a^	**rho^b^**

Vegetables	90	1.4 (1.2-1.6)	86	1.7 (1.5-2.0)	n.s.	0.74
Packaged Snacks	40	0.4 (0.3-0.5)	37	0.5 (0.3-0.6)	n.s.	0.60
Fruit	93	2.0 (1.8-2.4)	91	2.1 (1.8-2.4)	n.s.	0.66
Chocolate/Confectionary	39	0.4 (0.3-0.5)	32	0.4 (0.3-0.6)	n.s.	0.57
Cake/Sweet Biscuits	53	0.6 (0.5-0.8)	57	0.7 (0.6-0.9)	n.s.	0.60
Water	99	3.2 (2.8-3.5)	94	4.0 (3.6-4.4)	0.001	0.59
Fruit Juice	49	0.6 (0.4-0.8)	42	0.9 (0.6-1.2)	n.s.	0.88
Cordial/Soft Drink	36	0.6 (0.4-0.8)	32	0.7 (0.4--0.9)	n.s.	0.82
Plain Milk	82	1.7 (1.4-1.9)	81	2.1 (1.7-2.4)	n.s.	0.65
Flavoured Milk	21	0.3 (0.2-0.5)	22	0.6 (0.3-0.8)	n.s.	0.74

^a ^Kruskal Wallis test between EPAQ and 24-hr recall; n.s. not significant; ^b^Spearman correlations (rho) between 24 hr-recall and EPAQ, all p < 0.001

The energy density (energy per gram) of the food/beverage groups as derived from the 24 hr-recall data was as follows (mean ± standard deviation): vegetables 3.2 ± 0.2, packaged snacks 18.5 ± 0.9, fruit 2.8 ± 0.3, chocolate/confectionary 15.9 ± 0.9, cake/sweet biscuits 16.5 ± 0.6, fruit juice 1.6 ± 0.04, cordial/soft drink 1.3 ± 0.1, plain milk 2.6 ± 0.1, flavoured milk 3.1 ± 0.1.

## Discussion

The aim of this study was to determine the relative validity of the Eating and Physical Activity Questionnaire (EPAQ) compared to 24 hr-recall for children aged two to five years. The EPAQ produced good relative validity for estimating intake of the obesity-related key foods and beverages examined. This study demonstrates that this simple survey can be used for population monitoring and evaluation of obesity prevention programs, where estimates of average population intakes are often needed.

Although the survey performed well, when using the EPAQ parents tended to underestimate their child's consumption of beverages and during the interviews it became clear that many parents did not include milk on cereal and did not always identify flavoured milk (eg. adding honey to plain milk) when completing the EPAQ. In addition, parents stated during interviews that water consumption was difficult to quantify because many children had access to water bottles and dispensers where they could refill their own drink bottles, both at home and while in care or preschool. To assist with improved reporting of milk the example of "including milk on cereal" has now been added to the EPAQ to prompt parents to consider this in addition to other servings.

The correlations found in this study between 24 hr-recall and the EPAQ are similar to other validity studies comparing foods and food groups between food frequency questionnaires, dietary records or recall methods. In these previous studies, correlations ranged from 0.17 [29,30] to 0.78 [30] but were generally in the range of 0.40 to 0.50 [21,29,18,38]. In addition, the correlations between the two methods for beverages were found to be higher than those of the food items, a similar finding to other studies [31,39,40]. The advantage of using the EPAQ for population-level data is that it is specific to obesity and early childhood (a target group currently lacking specific research tools), is relatively low cost to administer, has relatively low participant burden, and is simple to analyse.

The energy density of the foods and beverages was also determined in this study. Compared to other known energy densities of these food groups [27] the energy density of fruit juice was lower in this study, which relates to the fact that parents of young children frequently diluted the fruit juice before giving it to their children. Also, the energy density of the vegetables category were relatively high for vegetables [27] which reflected the fact that parents included fried potato (fries) within this category. If the intake of all vegetables other than fried potato was specifically of interest, we recommend including a statement to this effect on the survey to guide those completing the EPAQ not to count fried potato when estimating intake of this food group.

### Study strengths and limitations

The current study has several limitations. The methods relied on parental report of child intakes and some children spent time in care on the day of interest, however, parents have knowledge of the menus in child care centres and/or provide the foods for their child's consumption while they are in care or preschool. Importantly, the two methodologies used within this study both relied on parent's memory, and therefore have similar reporting errors, related to the parent's ability to specifically recall the intake of the study child, their knowledge of the total intake of the child on the day of interest and social desirability bias. Also, the study population was predominantly Caucasian with a bias towards families of a higher socioeconomic status; therefore the questionnaire needs to be validated in other cultural groups and in disadvantaged families.

## Conclusion

The EPAQ is a simple and useful population-level tool for assessing the intake of obesity-related foods and beverages of children aged two to five years. When compared with 24 hr-recall food intake data, the EPAQ produced a good level of relative validity and the questionnaire has application for population monitoring and the evaluation of population-based obesity prevention public health interventions directed towards young children and their families.

## Competing interests

The authors declare that they have no competing interests.

## Authors' contributions

CB collected the data, performed the analysis and prepared the first draft of the manuscript. AdS supervised the data collection, directed the data analysis, interpretation of findings and manuscript preparation. MN, AB and BS conceived the study, developed the study design and protocols. MN assisted with data collection and interpretation of findings. All authors critically reviewed the drafts and read and reviewed the final manuscript.
